# A Sensor Employing an Array of Silicon Photomultipliers for Detection of keV Ions in Time-of-Flight Mass Spectrometry

**DOI:** 10.3390/s25051585

**Published:** 2025-03-05

**Authors:** Antonio Mariscal-Castilla, Markus Piller, Jerome Alozy, Rafael Ballabriga, Michael Campbell, Oscar de la Torre, David Gascón, Sergio Gómez, David Heathcote, Joan Mauricio, Dennis Milesevic, Andreu Sanuy, Claire Vallance, Daniel Guberman

**Affiliations:** 1 Department of Física Quàntica i Astrofísica, Institut de Ciències Del Cosmos, Universidad de Barcelona, 08007 Barcelona, Spain; 2 Department of Microelectronics, Cern, Esplanade des Particules 1, 1211 Meyrin, Switzerland; 3 Serra Hunter Fellow, Department of Enginyeria Electrònica, Universitat Politècnica de Catalunya, 08034 Barcelona, Spain; 4 Chemistry Research Laboratory, Department of Chemistry, University of Oxford, Oxford OX1 3TA, UK

**Keywords:** silicon photomultiplier, SiPM, time-of-flight mass spectrometry, ion detector, ASICs, velocity-map imaging

## Abstract

Pixellated scintillation detectors have the potential to overcome several limitations of conventional microchannel-plate-based detectors employed in time-of-flight mass spectrometry (ToF-MS), such as extending detector lifetime, reducing vacuum requirements, or increasing the ion throughput. We have developed a prototype comprising a fast organic scintillator (Exalite 404) coupled to an array of 16 silicon photomultipliers (SiPMs), with read-out electronics based on the FastIC application-specific integrated circuit (ASIC). Each SiPM signal processed by FastIC is fed into its own time-to-digital converter (TDC). The dead time of a single channel can be as short as ∼20 ns. As a result, our system have the potential to process ion rates above $10^{9}$ cm^−2^ s^−1^. We have evaluated the performance of our prototype using a velocity-map imaging ToF-MS instrument, recording the time-of-flight mass spectra of C_3_H_6_ and CF_3_I samples. We achieved time resolutions of (3.3±0.1) and (2.5±0.2) ns FWHM for ions of mass-to-charge ratio (m/z) values of 196 and 18, respectively. This corresponds to a mass resolution of ∼1000 for m/z<200, which we found to be dominated by the spread in ion arrival times.

## 1. Introduction

Scintillators coupled to fast detectors are widely used in several fields and applications, such as high-energy physics [1], medical imaging [2], dosimetry [3], and archaeology [4]. Over the last seventy years, various researchers have studied the possibility of using this type of detection system in time-of-flight mass spectrometry (ToF-MS; see, for instance, [5,6,7,8]), and scintillators are now finding their way into the ion detectors of state-of-the-art commercial time-of-flight instruments [9,10,11]. In most of these detectors, the ions hit a microchannel plate (MCP) detector or a conversion dynode, generating secondary electrons, which are accelerated towards the scintillator. In this way, more scintillation photons are produced, and the detection efficiency increases.

There have been a small number of investigations into the possibility of removing the MCPs and directly detecting the ions using a scintillator coupled to a photodetector [6,8,12]. This approach is widely used in the high-energy physics/astrophysics community (see, for instance, [13,14,15]) but, until recently, has not been suitable for the detection of low-energy ions due to the fact that the impact of a low-energy ion with a scintillator generally generates too few photons to be detected (∼10 photons/keV) [16,17,18]. The advent of faster, brighter scintillators [19,20,21], coupled with a new generation of photodetectors capable of single-photon detection [22,23,24], opens up new opportunities for the development of low-energy ion detectors based on this concept. Removing the MCPs from an ion detector has a number of advantages. While MCPs have outstanding time resolution, which is crucial in ToF-MS systems, they are expensive and fragile, suffer from ageing, and have strict high-vacuum requirements. They are also prone to saturation, with a maximum output current that limits the achievable data acquisition rate [25,26,27,28]. As noted above, eliminating the MCPs drastically reduces the number of photons available for detection, in principle compromising the detection efficiency, particularly for high-m/z ions. However, there are ways to overcome this issue. For instance, Dubois and coworkers [6] were able to increase the detection efficiency for 70 kDa ions by placing a copper grid immediately before the scintillator as a secondary ion converter. Later [7], the m/z range was increased to 90 kDa using a more complex method in which the scintillator was coated with a thin layer of aluminium (∼1000 Å) and the ion beam was directed towards it to generate secondary cations. These cations were then guided to a washer-shaped aluminium component to produce secondary electrons, which were subsequently accelerated to the scintillator.

Most scintillation detectors employed in ToF-MS applications are equipped with a photomultiplier tube (PMT) as a photodetector. Wilman et al. [8] investigated replacing this with a silicon photomultiplier (SiPM). SiPMs offer several advantages with respect to PMTs, including a higher photodetection efficiency (PDE), a greater degree of compactness and robustness, insensitivity to magnetic fields, and no requirement for a high operation voltage [29,30,31]. A single-photon time resolution (SPTR) below 100 ps full width at half maximum (FWHM) can be obtained with commercial SiPMs [32,33], and the time resolution can be as good as a few tens of ps for higher-intensity signals [34]. In addition, SiPMs can easily be grouped into compact arrays to achieve a few tens of channels per cm^2^, offering substantial possibilities for increasing the ion detection throughput by orders of magnitude via parallelisation of the detection system.

SiPM arrays are particularly well suited for use in imaging mass spectrometry and multi-mass imaging experiments, such as velocity-map and spatial-map imaging mass spectrometry (VMImMS) [35,36]. These techniques require fast position-sensitive detectors capable of operating at high acquisition rates to achieve 3D imaging (x,y,t) of ion velocity distributions. A camera based on SiPM technology offers an increase in speed of an order of magnitude or more relative to the fastest existing optical cameras for time-resolved particle imaging [37,38,39]. One of the main challenges associated with building such a camera is the requirement of signal acquisition and digitisation of several tens to hundreds of closely packed channels. Unsurprisingly, the cost and complexity of the detector increase with the number of readout channels. In this regard, application-specific integrated circuits (ASICs) offer a highly compact, power-efficient, and cost-effective solution [40,41,42,43,44,45].

In the following, we report results from the first ToF-MS experiments performed with an array of 16 SiPMs coupled to a fast scintillator to directly detect the ions without MCPs. The SiPMs were read out using two FastIC ASICs. FastIC is an eight-channel ASIC developed for fast-timing applications and easily scalable for read-out of hundreds of pixels [45,46,47].

## 2. Materials and Methods

### 2.1. The FastIC ToF-MS Prototype

**Figure 1:**
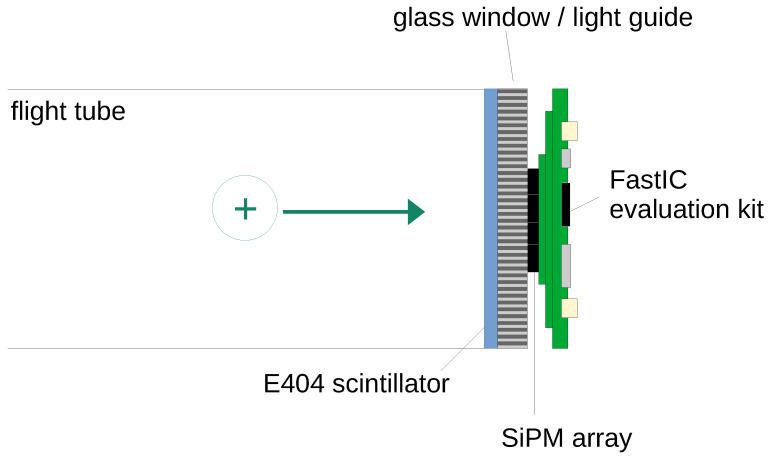
The key components of the FastIC ToF-MS prototype detector.

**Figure 2:**
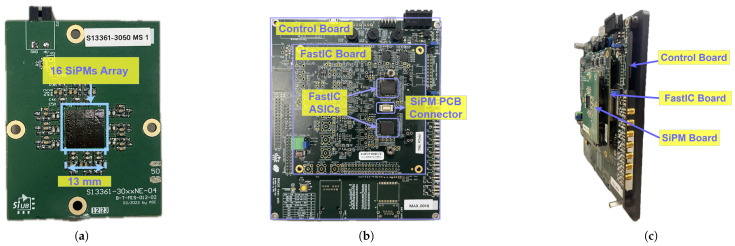
(**a**) PCB containing the 16-SiPM array; (**b**) FastIC evaluation kit; (**c**) side view of the SiPM array connected to the FastIC evaluation kit.

The detector we have developed is shown schematically in Figure 1 and consists of an Exalite 404 (E404) scintillator (see [12,19] for details) coupled to the SiPM sensor array and readout system. Using vacuum sublimation, the E404 scintillator is deposited onto the vacuum side of a 40 mm diameter fibre-optic plate (Photonis, France) pre-coated with indium tin oxide (ITO) [12,19]. This fibre-optic plate serves as the interface between the vacuum chamber and the exterior. The scintillator emission peaks at ∼412 nm, with a scintillation light yield of ∼50 photons/keV [19], a rise time of ∼1.1 ns, and a decay time of ∼1.5 ns [38]. Incoming ions strike the scintillator with energies of a few keV, typically generating a few scintillation photons per ion. The array of 16 SiPMs (S13361-3050-NE04 from Hamamatsu Photonics K.K, Hamamatsu, Japan—see Figure 2a) is mounted outside the vacuum chamber on the output face of the fibre-optic plate and is optically coupled to the plate using BC-630 silicone optical grease. Signals from the SiPM array are digitised and read out using a FastIC evaluation kit. Each SiPM has an area of 3 × 3 mm^2^ and is protected with an epoxy resin (refraction index $n_{D}≃$ 1.55). The SiPMs have a breakdown voltage of ∼52 V at room temperature. They achieve a peak PDE of ∼55% at 450 nm when operated at 7 V above breakdown. The total area occupied by the array, including dead spaces between SiPMs, is 13 × 13 mm^2^. Each SiPM has a fill factor of 74%, resulting in ∼106.6 mm^2^ total sensitive area of the array employed, which represents ∼8% of the total scintillation area.

The FastIC evaluation kit comprises two printed circuit boards (PCBs): one that houses two FastIC ASICs and another equipped with a field-programmable gate array (FPGA) (refer to Figure 2b). FastIC is an 8-channel ASIC designed to process signals from high-gain detectors, including SiPMs, PMTs, and MCPs, specifically for fast-timing applications. The ASIC processes incoming pulses, allowing the extraction of crucial information regarding their arrival time and amplitude. The arrival time information is obtained using a threshold comparator. The level of the comparator can be modified by the user. More details on the ASIC can be found in [46,47].

**Figure 3:**
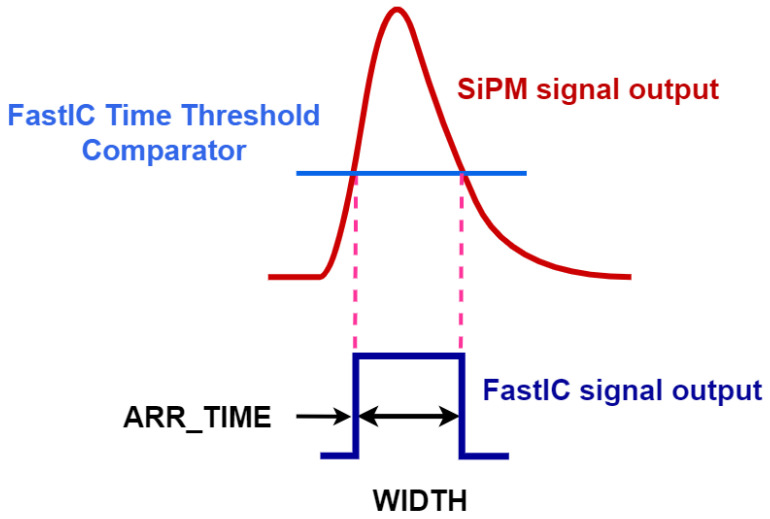
Scheme showing how FastIC’s high-rate mode processes SiPM signals. FastIC produces a single binary pulse. The rise time of this binary pulse gives the arrival time of the signal (ARR_TIME). The pulse width (WIDTH) is given by the signal time over threshold and is thus related to the signal amplitude.

The ASIC has two operation modes: ‘standard’ and ‘high-rate’. In the standard mode, FastIC outputs two consecutive binary pulses: the first one encodes in its rise time the arrival time of the input signal, while the width of the second one is proportional to the amplitude of the input signal. The main drawback of this mode is that it has a dead time of 500 ns per detector channel. In the high-rate mode, FastIC outputs a single binary pulse that encodes in its rise time the arrival time of the input signal, while the width of this binary pulse is related non-linearly to the amplitude of the input signal (see Figure 3). This mode, which is the one employed in the experiments reported here, results in a channel dead time as short as ∼20 ns. In Section 3, we will show that it can provide information about the intensity of the incoming signals for fluxes of a few photoelectrons (phes).

The binary signals generated by FastIC are digitised by a multi-channel time-to-digital converter (TDC) implemented in the FPGA of the FastIC evaluation kit. In the current implementation, the TDC processes 17 signals: 16 from the two FastIC ASICs and one additional trigger signal. For each FastIC signal, the TDC outputs two values, which we labelled ARR_TIME (the arrival time of the recorded signal; see Figure 3) and WIDTH (related to the amplitude of the signal).

### 2.2. Characterisation of the Detector

Initially, the SiPM/FastIC detection and readout system were characterised using pulses from a picosecond pulsed laser before being coupled with the fast scintillator and incorporated into a custom-built velocity-map imaging mass spectrometer for ToF-MS measurements. These two steps are described separately in the following.

#### 2.2.1. Optical Characterisation of the SiPM Array

One of the most important design specifications for a ToF-MS detector is its intrinsic time resolution. In our case, the overall time resolution of the detector comprises contributions from the time responses of the scintillator, the SiPM, and the readout electronics. The time response of the E404 scintillator has been characterised previously [38]. In the present work, we characterised the SPTR of the SiPM array readout using the FastIC evaluation kit. These measurements were performed by illuminating the SiPM array directly with pulses of light from a picosecond pulsed laser (picosecond laser diode system ‘PiL040X’ from Advanced Laser Diode Systems A.L.S. GmbH, Berlin, Germany). The laser generates ∼405 nm (∼1 nm FWHM), ∼28 ps pulses. The laser output was coupled via a single-mode optical fibre to a collimator, before entering a liquid-crystal beam attenuator (LCC1620 from Thorlabs, Newton, NJ, USA). This way, we could achieve a photon flux of a few photons at the front of the SiPM array.

The SiPMs were operated at a bias voltage of 59 V. In these measurements we set the FastIC discriminator threshold below the single-phe level. During the analysis, we applied cuts in the WIDTH values to select single-phe events. From the resulting subsample, we extracted the ARR_TIME values and built a distribution that we could fit to an exponentially modified Gaussian (EMG) function, which is standard in SPTR studies with SiPMs [32]. Typically, the SPTR is defined as the FWHM of this fitted distribution, which includes contributions from the laser, the SiPM, and the electronic readout. To obtain the intrinsic SPTR of the photonic detector, the contribution of the laser must be deconvoluted.

#### 2.2.2. Characterisation of Ion Detector Performance Within a ToF-MS Experiment

The complete detector described in Section 2.1 was mounted within a custom-designed multi-mass velocity-map imaging instrument located at the University of Oxford. The instrument is essentially a time-of-flight mass spectrometer that is designed to record images of the scattering or velocity distributions of each ion in addition to its time of flight. The specific details of the instrument have been described previously [48,49], and will only be summarised fairly briefly in the following.

**Figure 4:**
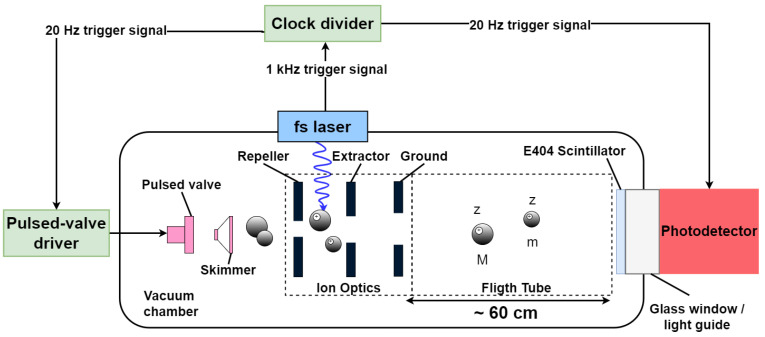
A schematic illustrating the main components of the custom-built velocity-map imaging ToF-MS instrument.

The ion detector was used to record ion signals arising from the dissociative photoionisation of CF_3_I and C_3_H_6_. Both molecules were prepared in a seeded supersonic molecular beam using helium as the buffer gas. The supersonic expansion was generated using a Parker Hannifin Series 9 pulsed solenoid valve operating at a frequency of 20 Hz and passed through a skimmer before entering the interaction region through a 2 mm hole in the repeller plate of the velocity-mapping ion optics assembly (see Figure 4). The complete ion optics assembly comprised three electrodes, referred to as the repeller, extractor, and ground plates, respectively. Within the interaction region between the repeller and extractor electrodes, the skimmed molecular beam was intersected at right angles by a high-intensity 800 nm, ∼65 fs pulsed laser beam from a Spectra-Physics Solstice Ti:Sapphire laser. The 1 kHz laser pulses were synchronised with the 20 Hz molecular beam pulses by triggering the pulsed valve from the laser trigger via a clock divider. The trigger signal was also sent to the FastIC module.

Interaction with the laser pulses initiated ionisation and fragmentation of the CF_3_I or C_3_H_6_ molecules in the molecular beam, generating a variety of fragment ions. The electric field maintained within the velocity-mapping ion optics assembly accelerated the ions along a ∼60 cm field-free linear time-of-flight tube to the ion detector, mapping the three-dimensional velocity distributions of the nascent ions into a two-dimensional projection on the front surface of the detector. Of particular note, parent molecular ions that do not undergo fragmentation retain the extremely narrow transverse velocity distribution of the supersonic molecular beam and are mapped onto a small spot in the centre of the detector. In contrast, dissociation events impart momentum and kinetic energy to the fragments, and fragment ions therefore exhibit characteristic scattering distributions that reflect the forces acting during the dissociation event. Due to our limited number of pixels and spatial resolution, we were not able to measure the fragment velocity distribution, which, regardless, was not our scope. Our purpose was to study the feasibility of using arrays of SiPMs to detect the accelerated ions and evaluate their time resolution, as in a conventional ToF-MS instrument.

We focused the parent ion beam onto the central pixels of the SiPM array and conducted the experiments under conditions similar to those used with an MCP-based detector, specifically a laser pulse energy of approximately 150 μJ and an acceleration potential of 7 kV. We acquired ∼4 k and ∼6 k thousand ToF cycles for the CF_3_I and C_3_H_6_ molecules, respectively. Additionally, the SiPMs were biased at 59 V, and the FastIC time discriminator threshold was set to be able to record the single-phe signal.

Time distributions for individual ions were fitted to an EMG function, and the time resolution was defined as the FWHM of this function. To determine the mass resolution, we employed the equation $R=\frac{m}{\Delta m}=\frac{t}{2\Delta t}$ [50].

## 3. Results

### 3.1. SiPM Array Single-Photon Time Resolution

**Figure 5:**
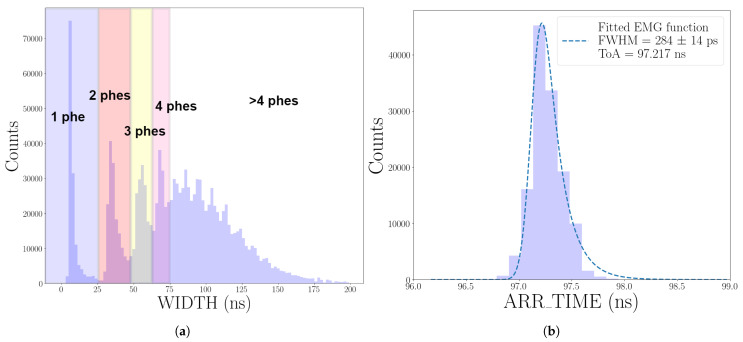
Example of the distributions obtained after an SPTR measurement with one channel of the SiPM array. (**a**) WIDTH distribution of all the events. (**b**) ARR_TIME distribution for single-phe events.

Figure 5 shows the results of an SPTR measurement for one of the channels of the 16-SiPM array, recorded as described in Section 2.2.1. Here, ARR_TIME is the time difference between the trigger from the laser driver and the time measured by FastIC. We first consider the WIDTH distribution shown in Figure 5a). The first four peaks correspond to events of one (peak at ∼6 ns), two (∼34 ns), three (∼55 ns), and four (∼68 ns) phes. The peaks are harder to distinguish when the signal is larger due to the non-linear nature of the FastIC operation mode employed (see Section 2.1). Figure 5b) shows the ARR_TIME distribution of single-phe events, fitted with an EMG function. The SPTR obtained, defined as the FWHM of the fitted function, was (284±14) ps. This result includes all contributions to the time resolution (laser, SiPM, and read-out electronics). However, in this case, the contribution from the laser, which has a pulse width of a few tens of ps, is negligible.

**Figure 6:**
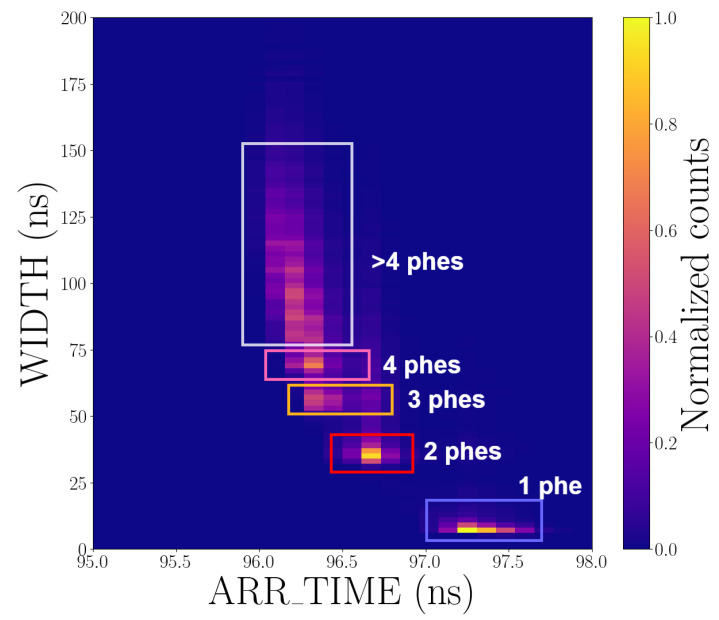
Two-dimensional histogram of WIDTH and ARR_TIME values for one channel with the SiPM array biased at 59 V.

Figure 6 shows a two-dimensional histogram of the WIDTH and ARR_TIME values, revealing a correlation between the ARR_TIME and WIDTH, namely, that the mean time of arrival (ToA) is longer for smaller signals. This *time-walk* effect is intrinsic to systems that employ a leading-edge discriminator to extract arrival times [51]. When required, simple correction algorithms can be applied to compensate for this effect [46,52,53]. The correlation plot also shows that the spread of arrival times is larger for weaker signals. As a result, the time resolution of SiPMs typically improves with the intensity of the incoming flux.

### 3.2. ToF-System Time Resolution

**Figure 7:**
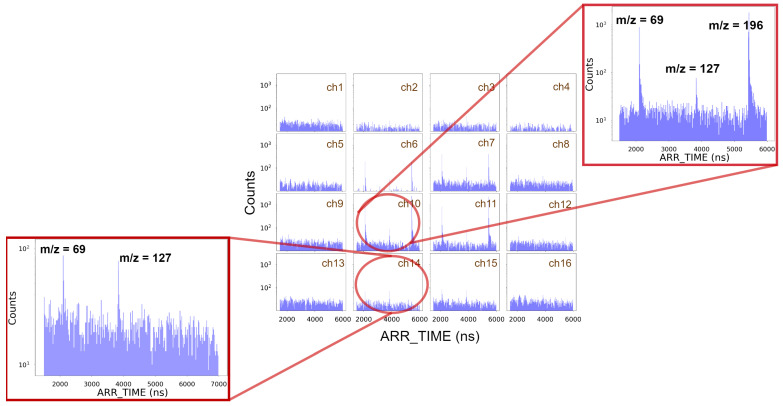
Example of a reconstructed spectrum of CF_3_I after ∼4 k ToF cycles.

Figure 7 shows an example of the CF_3_I photofragment ion time-of-flight spectra recorded by each SiPM. Here, ARR_TIME is the time difference between the trigger from the clock divider and the time measured by FastIC. The three observed peaks correspond to the fragment ions ${\mathrm{CF}}_{3}^{+}$ (m/z=69) and I^+^ (m/z=127) and the parent ion CF_3_I^+^ (m/z=196). The parent molecular ion is detected only in the central channels (6, 7, 10 and 11) because it retains the narrow transverse velocity distribution of the supersonic molecular beam, as explained in Section 2.2.2. On the other hand, the fragment ions show a wider spatial distribution (see channels 14 and 15), especially the I^+^ fragment, due to their characteristic scattering distributions resulting from the dissociation event [54,55]. As pointed out in Section 2.2.2, the FastIC time comparator threshold was set to detect the single-phe signal. Thus, the background shown in Figure 7 can be attributed to SiPM optical noise, namely, dark counts and cross-talk. We attribute the differences in background between channels (e.g., between channels 6 and 1) to variations in the FastIC time comparator thresholds across channels.

**Figure 8:**
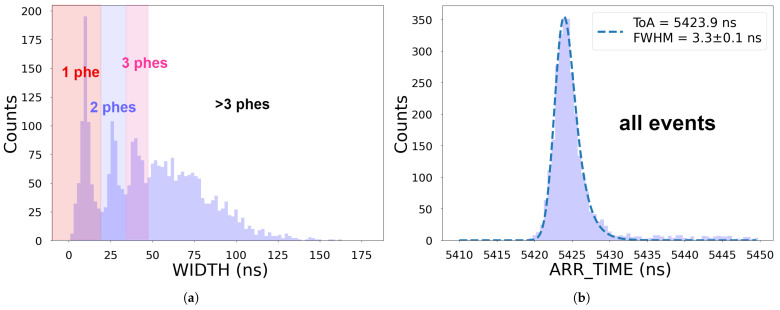
WIDTH (**a**) and ARR_TIME (**b**) distributions of the CF_3_I^+^ peak recorded on channel 10.

Figure 8 shows the ARR_TIME and WIDTH distributions recorded on channel 10 for the CF_3_I^+^ peak. In the WIDTH distribution, the peaks corresponding to one-, two, and three-phe events can be distinguished (see Figure 8a). This implies that a typical signal collected in a single cycle consists of a few photons. Note that the intensity of the detected flux depends on many parameters, including the number of ions arriving at the scintillator, the number of photons produced in the scintillator, the collection efficiency, and the SiPM PDE. We fitted the ARR_TIME distribution to an EMG function and calculated the FWHM of the fitted function, finding a time resolution of (3.29±0.07) ns (see Figure 8b), which is compatible with a mass resolution ($R=\frac{m}{\Delta m_{FWHM}}$) of ∼1200. The time resolution is affected by the time spread with which the ions reach the detector, the scintillator properties (light yield and rise and decay times) and the intrinsic time resolution of the photodetector (including the electronics).

**Figure 9:**
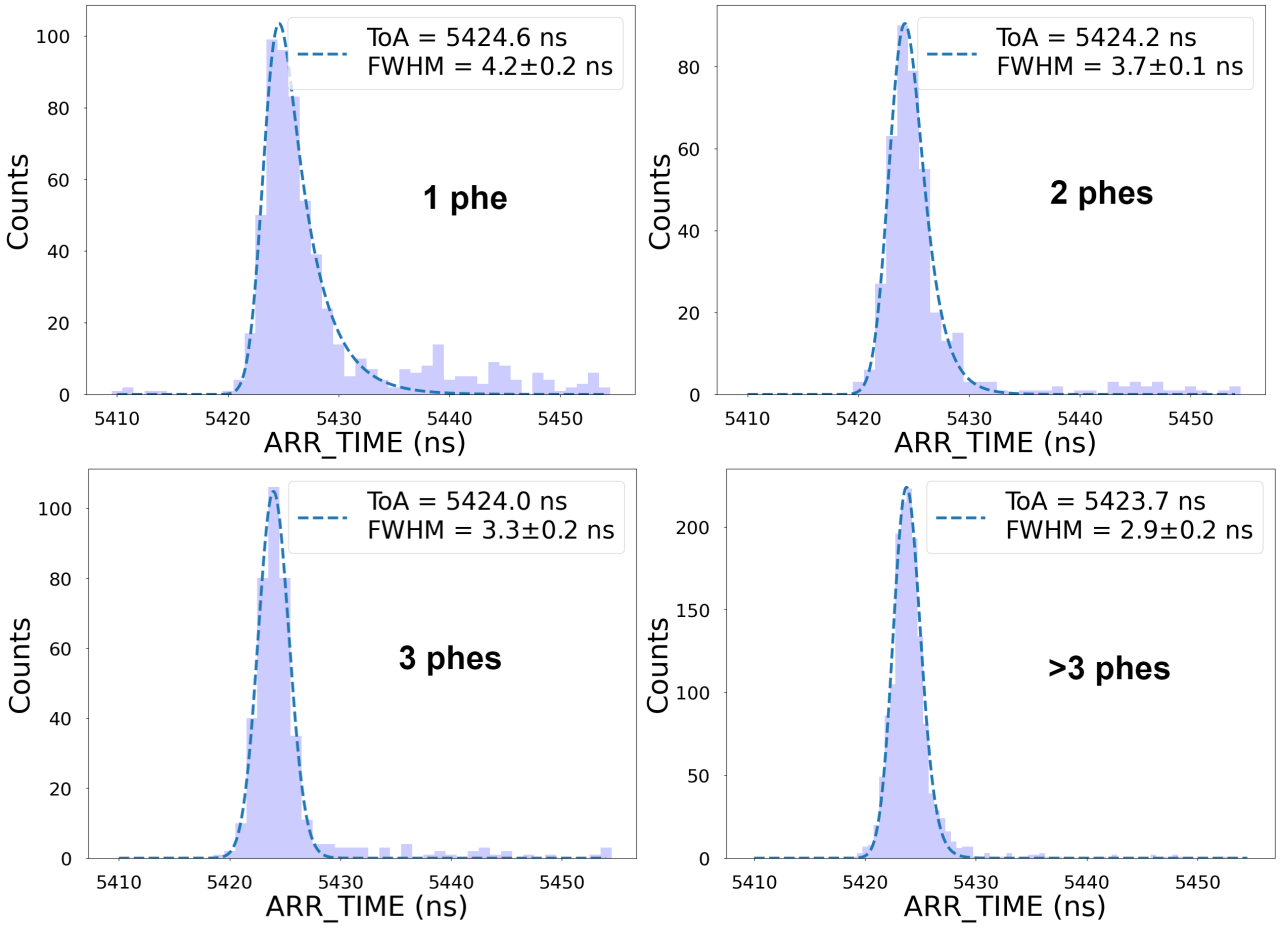
Arrival time distribution of the CF_3_I^+^ peak recorded on channel 10, when selecting events with WIDTH values corresponding to 1-phe, 2-phe, 3-phe, and >3-phe events.

**Figure 10:**
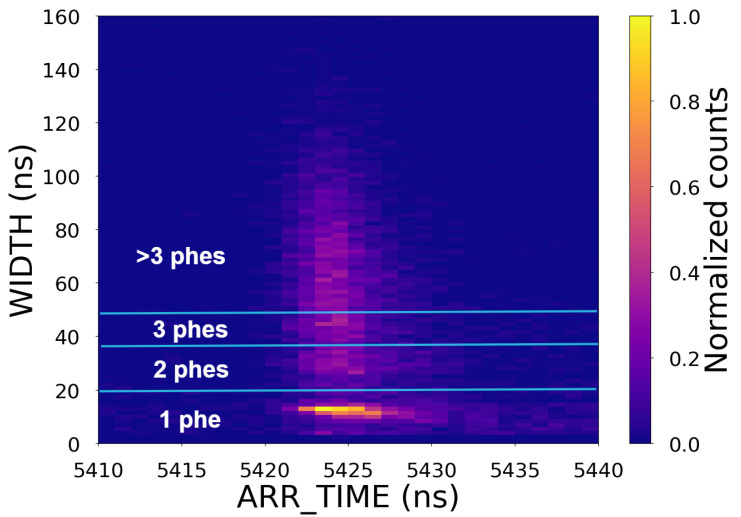
Two-dimensional histogram of ARR_TIME and WIDTH for CF_3_I^+^ and channel 10.

Figure 9 compares the arrival time distribution of the same CF_3_I^+^ peak after applying different cuts in WIDTH to select events of one, two, three, and more than three phes, showing that time resolution improves for larger signals. This is primarily because the time jitter associated with the scintillator–photodetector pair decreases as the number of detected photons increases [56,57]. The expected jitters associated with the scintillator and the photodetector (including electronics) for single-phe events are ∼2 ns [38] and ∼284 ps (see Section 3.1), respectively. The fact that we found a time resolution larger than 4 ns for single-phe events (top left panel in Figure 9) suggests that the time resolution of the system must be dominated by the spread in the arrival time of the ions. This spread is much larger than the typical spread associated with the time-walk effects described in Section 3.1, making any time-walk correction unnecessary. This can be seen in Figure 10, which shows the measured WIDTH as a function of ARR_TIME and can be compared with the data shown in Figure 6.

**Figure 11:**
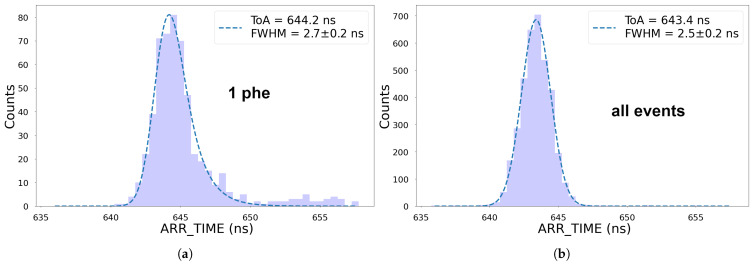
Time resolution of a background peak associated with H_2_O^+^ for single-phe events (**a**) and all events (**b**). Data recorded on channel 10.

Figure 11 shows the ARR_TIME distribution associated with ions of m/z=18 (H_2_O^+^ background ions). The presence of this background signal is due to the residual gas present in the vacuum chamber. The time resolution found when filtering one-phe events was (2.7±0.1) ns FWHM, which is significantly lower than was found for CF_3_I^+^. This would have not been the case if the time resolution had not been dominated by the spread of the arrival time of the ions. At low m/z, the ion arrival time spread is narrower and even comparable to the expected jitter associated with the scintillator during one-phe events. The time resolution found when using all events was (2.5±0.1) ns FWHM, which corresponds to a mass resolution of ∼770.

**Figure 12:**
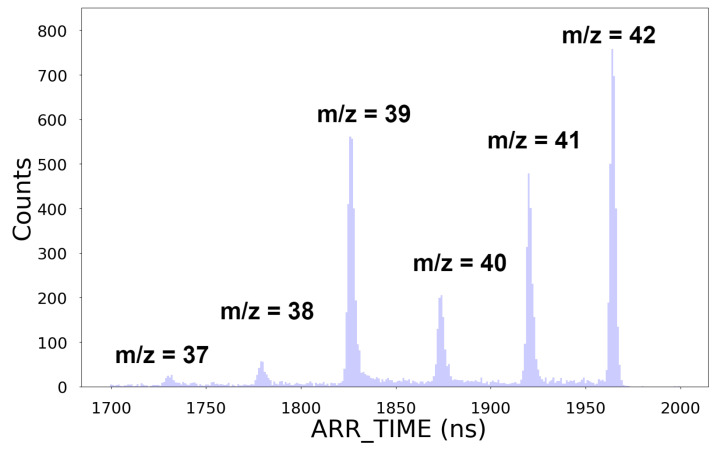
A reconstructed spectrum of propylene after ∼6 k ToF cycles recorded on channel 10.

As a second example, Figure 12 shows a reconstructed photofragment ion time-of-flight spectrum recorded for the molecule C_3_H_6_. All peaks from m/z=37 ($C_{3}H_{1}^{+}$) to m/z=42 ($C_{3}H_{6}^{+}$) can be clearly distinguished. The distance between consecutive peaks is ∼50 ns. We found a time resolution of (2.9±0.2) ns FWHM for m/z=42, which corresponds to a mass resolution of ∼730.

## 4. Discussion

We have described, for the first time, the implementation of an ion-to-photon detector for ToF-MS that employs an array of SiPMs. Despite the constraints imposed by the small photodetection area of our prototype and the limited mass resolution of the ToF-MS instrument employed, we were able to record and reconstruct the mass spectra of CF_3_I and C_3_H_6_ and to evaluate the time resolution of the system.

One of the key elements of our detector is the FastIC ASIC, which can process the signals of several SiPMs with compact electronics and low power consumption (∼12 mW/channel [46]). Our proof-of-concept prototype employed 16 SiPMs, but since we rely on an ASIC readout, the solution is relatively easily scalable to the hundreds of pixels that would be needed to cover the whole scintillator area [45]. This will become even more straightforward with the release of the next version of FastIC (named FastIC+), which has its own on-chip TDC. A pixellated detector, in which each channel has its own TDC, offers several advantages. One of these is the ability to deal with high ion rates. A detector such as the one we employed, equipped with 3 × 3 mm^2^ SiPMs, should be capable of handling ion rates higher than $10^{8}$ cm^−2^ s^−1^. Higher ion rates (>$10^{9}$ cm^−2^ s^−1^) could be processed if smaller SiPMs were used, at the expense of increasing the cost of the detector (since a larger number of SiPMs and FastIC ASICs per unit area would be needed). In this regard, the proposed detector should outperform traditional detectors based on MCPs, which can sustain ion rates up to ∼$10^{7}$ cm^−2^ s^−1^ [27,28]

A second advantage of our approach is that the detector does not have the stringent vacuum requirements associated with MCP-based detectors. Moreover, SiPMs are robust devices that do not suffer from ageing. They are also much more compact than the PMTs that are used in commercial ion-to-photon conversion detectors and do not require high voltages for operation. Taken together, these features open up possibilities for designing compact and portable ToF-MS instruments that could be useful in applications such as space exploration [58] or environmental monitoring [59].

Time resolution is a critical parameter for a ToF-MS instrument, since it directly determines the achievable mass resolution. As discussed above in Section 3.2, the time resolution achieved in the present experiments is mostly dominated by the spread in the arrival time of the ions at the scintillator. When the same VMImMS system is equipped with an MCP-based detector employing the Pixel Imaging Mass Spectrometry (PImMS) camera [37], the time resolution is dominated by the photodetector, which has a resolution of ∼25 ns. The ToF-MS instrument used in this study was optimised for imaging the ion velocity distribution, which came at the cost of reduced time resolution. By optimising the extraction potentials and/or modifying the ion lens design, it might be possible to improve the overall time resolution of the system. As a reference, another MCP-based detector that employed a high-resolution photodetector (Timepix3) and a slower scintillator (P47) achieved a time resolution of ∼4.7 ns FWHM [39].

One of the main limitations of our detector is its low light output. The scintillator has a typical light output of the order of 10 photons per keV of deposited energy, but the actual light output is reduced for low-energy particles due to scintillation quenching [60]. A significant fraction of the scintillation photons are also expected to be lost due to inefficiencies in the light collection. Finally, with a typical PDE of ∼50%, we only expect to detect about half of the photons that actually reach the SiPMs. Taken together, these factors explain why most of the signals we observed were events of a few photons. The low light output from the scintillator has a negative impact on the detection efficiency of the system, which, as shown in [6], could be critical at high m/z values. We were not able to measure the ion detection efficiency directly in our experiments, as we could not control with enough precision the number of ions hitting the scintillator per cycle. However, we do not expect it to be better than traditional detectors based on MCPs, for which the detection efficiency is mostly dominated by the dead space between the microchannels (typically ∼50% of the MCP area).

The low photon flux also limits the time resolution of the detector. The time resolution of a scintillator detector improves rapidly with the number of scintillation photons detected per event [56,57]. Considering the fast time response of the scintillator employed and the previous results obtained with FastIC and SiPMs [46], it should be possible to improve the detector time resolution down to a few tens of ps if we are able to increase the light output.

The intensity of the detected flux can be increased by improving the light collection efficiency. There are a number of ways in which this could be achieved, such as improving the optical coupling of the SiPMs to the exit window or exploring alternative to the traditional light guides. However, in order to obtain a significant boost in the light output, we would need to increase the number of scintillation photons generated per incident ion. This could be achieved by accelerating the ions to higher potentials, either within the ion optics or as they approach the detector, or by introducing elements such as converter plates or grids, or even an MCP, to convert the primary ions into secondary ions or electrons.

A camera based on SiPMs and FastIC holds considerable promise for a wide variety of applications in time-of-flight mass spectrometry. In conventional time-of-flight measurements, such a system has the potential to provide a considerable boost in ion throughput due to the large number of parallel time-of-flight detection channels. In velocity-map and spatial-map imaging time-of-flight measurements, it offers excellent time resolution competitive with the current state of the art [39], single-ion detection sensitivity, and scalable capabilities for recording velocity and/or spatial distributions for ions of interest, all without requiring the use of MCPs and with considerable scope for future improvements in all areas.

## Data Availability

The authors will share the data upon reasonable request.
